# Access to justice for Ethiopian migrant domestic workers: unveiling legal, structural, and gendered violence in Lebanon

**DOI:** 10.3389/fsoc.2024.1486769

**Published:** 2025-04-11

**Authors:** Banchi Yimer, Tsigereda Birhanu, Francisca Ankrah, Debora Asfaw, Omar Taleb, Jasmin Lilian Diab

**Affiliations:** ^1^Egna Legna Besidet, Beirut, Lebanon; ^2^Institute for Migration Studies, School of Arts and Sciences, Lebanese American University, Beirut, Lebanon

**Keywords:** migrant labor, justice, access, protection, legal frameworks, Kafala, Lebanon

## Abstract

This study investigates the barriers to legal access faced by Ethiopian migrant domestic workers (MDWs) in Lebanon, highlighting the legal violations they endure. The research encompasses all phases of criminal cases, from initial investigations to trial and verdict issuance. Utilizing a diverse methodology, the study draws on a sample of judgments from Egna Legna Besidet's Legal Program, qualitative interviews with 66 Ethiopian MDWs, and insights from 10 key informants and five legal experts. Ethical considerations, including a trauma-informed approach and thorough anonymization, were paramount. Findings reveal that MDWs encounter systemic injustices and insurmountable barriers within the Lebanese legal system, exacerbated by societal prejudices, exorbitant recruitment fees, language barriers, and limited legal awareness. The Kafala sponsorship system and restrictive immigration policies further hinder their legal navigation. Discriminatory attitudes, lack of legal safeguards, and inadequate support from embassies and consulates perpetuate a cycle of exploitation and abuse. The initial investigation stage is fraught with coercion and procedural violations, while trials and verdicts often lack fairness and adequate evidence. The study calls for comprehensive legal reforms, better enforcement of existing laws, accessible information, legal aid, and multi-stakeholder collaboration to dismantle these barriers and ensure justice for MDWs.

## 1 Introduction

Migrant domestic workers (MDWs) in Lebanon, estimated at 176,000 (Edwards and Sousa, 2024), face numerous complications in accessing justice, rooted in both systemic and societal issues (ILO, 2014; Amnesty International, 2019; Nasri and Tannous, 2017). These complications are multifaceted and deeply entrenched, creating formidable barriers that impede their pursuit of legal redress and protection. The Lebanese legal system presents significant challenges for MDWs seeking justice. The Kafala sponsorship system, which ties workers' residency to their employers, severely restricts their freedom and legal autonomy (Block et al., 2023; Pande, 2013). This dependency often deters MDWs from reporting abuses or seeking legal recourse due to fear of retaliation, job loss, or deportation (Diab et al., 2023). Additionally, the complex and opaque legal procedures further alienate MDWs, who may lack the necessary legal literacy to navigate these processes effectively (Saghieh, 2020; Rowitz, 2023).

This study investigates the systemic barriers to legal access faced by Ethiopian migrant domestic workers (MDWs) in Lebanon, with a focus on the legal violations they endure throughout various phases of criminal cases, from prosecution and initial investigations to trial and verdict issuance. The research seeks to answer key questions, including: (1) What legal and structural challenges do Ethiopian MDWs encounter when seeking justice for abuses in Lebanon? (2) How do the Kafala sponsorship system, discriminatory practices, and lack of legal awareness impact their ability to navigate the legal system? (3) What role do embassies, consulates, and civil society organizations play in supporting or hindering their access to justice? (4) How do these barriers intersect with gendered, racial, and socio-economic vulnerabilities to exacerbate their exploitation? Drawing upon a diverse range of data sources, including judgments compiled from Egna Legna Besidet's Legal Program, qualitative interviews with Ethiopian women who have undergone legal proceedings, key informants, and insights from attorneys and legal experts, the study provides a comprehensive examination of the barriers to justice faced by MDWs in Lebanon. By shedding light on these challenges, the research highlights the urgent need for structural reforms and robust protections to ensure the rights and dignity of MDWs are upheld.

## 2 Literature review

Language poses a significant hurdle for MDWs, most of whom do not speak Arabic fluently. The absence of translation services during legal proceedings and interactions with law enforcement exacerbates this issue, leaving MDWs unable to understand their rights, the charges against them, or the legal options available (The Legal Agenda ILO, 2020). This language barrier extends to their ability to communicate with legal representatives, further complicating their defense and access to justice (The Legal Agenda ILO, 2020). Deeply entrenched societal prejudices and discriminatory attitudes toward MDWs, particularly those from African countries, permeate various levels of the justice system. These biases often manifest in the form of mistreatment, neglect, and lack of empathy from law enforcement officers, legal professionals, and even judges (Ghaddar et al., 2020; Bondakji, 2021). MDWs frequently report experiences of racism, sexism, and xenophobia, which undermine their credibility and result in biased judicial outcomes (Pande, 2018).

The financial burden of accessing legal representation is a significant barrier for MDWs. Many cannot afford to hire lawyers, pay court fees, or cover the costs associated with legal proceedings (The Legal Agenda ILO, 2020). This economic disadvantage leaves them vulnerable to exploitation and without adequate support when facing legal challenges (Sater, 2013). The lack of state-sponsored legal aid specifically tailored to MDWs exacerbates this issue, leaving them without recourse. MDWs often lack awareness of their legal rights and the mechanisms available to seek redress (Abdulrahim et al., 2024). This lack is compounded by the isolation many experience due to restrictive working conditions and limited social networks (Pande, 2012). Without knowledge of their rights or how to access legal assistance, MDWs remain trapped in cycles of abuse and exploitation, unable to advocate for themselves effectively.

The support from home country embassies and consulates in Lebanon is often insufficient. MDWs report being neglected or ignored when seeking assistance, especially in legal matters (Diab et al., 2023). The embassies' limited capacity and lack of political will to provide timely and effective support leaves MDWs without crucial advocacy and protection, further hindering their ability to access justice (Barakat, 2023). The fear of retaliation from employers, who hold significant power over MDWs' legal and economic status, is a pervasive deterrent to seeking justice (Diab et al., 2023). Threats of deportation, physical harm, and further abuse are common, creating a climate of fear that silences many MDWs (Diab et al., 2023). This fear is compounded by the lack of protective measures and support systems for those who dare to come forward (El Hage, 2023).

The Lebanese legal system's structural inefficiencies, including prolonged detention periods, administrative delays, and lack of proper oversight, disproportionately affect MDWs (Ebert, 2015; Karam, 2024). Cases involving MDWs are often handled with less urgency and care, resulting in extended periods of detention without trial, inadequate legal representation, and unfair verdicts. The lack of accountability and oversight within law enforcement agencies further perpetuates these injustices (Mansour and Daoud, 2010; Malaeb, 2018). MDWs' isolation within Lebanese society, driven by cultural differences and restrictive employment conditions, exacerbates their vulnerability. This isolation limits their ability to seek help, access information, and connect with support networks.

## 3 Methodology

The methodology employed in this study was comprehensive and multifaceted, integrating diverse data sources and adhering to rigorous ethical standards throughout the research process. Primary data collection involved an exhaustive review of over 100 case files related to Migrant Domestic Workers (MDWs) and conducting in-depth qualitative interviews with 66 Ethiopian MDWs living in Lebanon. To complement these efforts, key informant interviews were held with 10 stakeholders, including representatives from government agencies, academic institutions, civil society organizations, UN agencies, and international humanitarian organizations. Furthermore, consultations were conducted with three legal experts and two lawyers who have extensive experience representing MDWs in legal proceedings. These combined approaches enabled the research to provide a holistic understanding of the systemic challenges Ethiopian MDWs face in accessing justice in Lebanon. For a demographic breakdown of the sample, see Tables 1–3.

**Table 1:** Demographic characteristics of the sample.

**Category**	**Details**	**Number of participants**	**Percentage**
Age at migration	Underage	12	18.18%
	18–40	43	65.15%
	Not reported	8	12.12%
	Could not recall	3	4.55%
Passport age accuracy	Accurate	10	15.15%
	Inaccurate	51	77.27%
	Did not remember	1	1.52%
	Not reported	4	6.06%
Duration in Lebanon	< 2 years	1	1.52%
	< 5 years	24	36.36%
	< 10 years	29	43.94%
	>10 years	9	13.64%
	Not reported	3	4.55%
Language proficiency	Arabic	52	78.79%
	English	2	3.03%
	Arabic & English	9	13.64%
	Multilingual	2	3.03%
Pre-migration occupation	Studying	25	37.88%
	Working	23	34.85%
	Both studying and working	4	6.06%
	Neither	1	1.52%
	Not reported	6	9.09%
Education level	College degree	4	6.06%
	High school	30	45.45%
	Elementary	15	22.73%
	Not reported	17	25.76%
Children in Lebanon	Had children	5	7.58%
	Did not have children	47	71.21%
	Not reported	14	21.21%
Legal documentation for offspring	Faced challenges	3	60% of those with children
	Did not report	2	40% of those with children

**Table 2:** Travel and migration experience.

**Category**	**Details**	**Number of participants**	**Percentage**
Travel arrangements	Recruitment agencies	50	75.76%
	Family members	4	6.06%
	Friends	5	7.58%
	Other	7	10.61%
Payment of fees	Ethiopia	28	42.42%
	Transit country	4	6.06%
	Lebanon	9	13.64%
	Ethiopia & Lebanon	18	27.27%
	Ethiopia, transit country & Lebanon	1	1.52%
	None	5	7.58%
Modes of entry	Direct flight	41	62.12%
	Through Sudan	9	13.64%
	Through Yemen	5	7.58%
	Through Dubai	4	6.06%
	Other	5	7.58%
	Not reported	2	3.03%
Knowledge of recruitment agents	Knew name of agent/agency	4	6.06%
	Forgot name	2	3.03%
	Knew Ethiopian agent/family member	2	3.03%
	Did not respond	3	4.55%
	Did not know	55	83.33%
Transit experiences	No proper bed	19	79.17% of those in transit
	Proper bed	2	8.33% of those in transit
	Did not report	3	12.50% of those in transit
	No physical abuse	21	87.50% of those in transit
	Verbal abuse	6	25% of those in transit
	Insufficient food	18	75% of those in transit
	Sexual harassment	1	4.17% of those in transit
	Did not report	3	12.50% of those in transit
Work locations	Beirut	23	34.85%
	Outside Beirut	26	39.39%
	Multiple locations	3	4.55%
	Unknown	8	12.12%
	Not reported	7	10.61%
Briefed on rights	Yes	3	4.55%
	No	61	92.42%
	Not reported	2	3.03%
Briefed on duties	Yes	4	6.06%
	No	60	90.91%
	Not reported	2	3.03%
Contract translation	Translated into native language	3	4.55%
	Not translated	60	90.91%
	Not reported	3	4.55%

**Table 3:** Working conditions.

**Category**	**Details**	**Number of participants**	**Percentage**
Monthly salary	< $150	8	12.12%
	$150	46	69.70%
	$200	7	10.61%
	$250	3	4.55%
	$400	1	1.52%
	Not reported	1	1.52%
Full payment from first employer	Received full payment	13	19.70%
	Did not receive full payment	47	71.21%
	Non-payment from multiple employers	4	6.06%
	Not reported	2	3.03%
Wages owed by employers	< 6 months' wages	29	43.94%
	< 1 year's wages	12	18.18%
	< 2 years' wages	4	6.06%
	< 3 years' wages	3	4.55%
	Fully paid	7	10.61%
	Not reported	10	15.15%
Payment arrangements	Sent to family	47	71.21%
	Paid directly in cash	2	3.03%
	Paid to agency	1	1.52%
	Not reported	16	24.24%
Decision on payment method	Decided by sponsor	43	65.15%
	Decided by participant	7	10.61%
	Not reported	16	24.24%
Living conditions	Sufficient food and water	19	28.79%
	Insufficient food and water	46	69.70%
	Not reported	1	1.52%
	Own bedroom	13	19.70%
	No private bedroom	50	75.76%
	Privacy	10	15.15%
	No privacy	51	77.27%
Household size	< 5 members	24	36.36%
	5–10 members	32	48.48%
	>10 members	1	1.52%
	Not reported	9	13.64%
Discrimination	Experienced discrimination	36	54.55%
Communication with family	Allowed	48	72.73%
	Not allowed	15	22.73%
	Charged for communication	1	1.52%
	Not reported	2	3.03%
Phone ownership	Allowed own phone	4	6.06%
	Not allowed	60	90.91%
	Occasionally allowed	1	1.52%
	Not reported	1	1.52%
Weekly day off	Allowed	3	4.55%
	Not allowed	63	95.45%
Leaving the house	Allowed	11	16.67%
	Not allowed	54	81.82%
	Not reported	1	1.52%

The data collection process, conducted over 4months, was structured around several key themes. These included the legal challenges encountered during prosecution, experiences of abuse both in domestic and legal contexts, interactions with law enforcement and judicial personnel, awareness of legal rights, and the role of embassies and recruitment agencies in facilitating or obstructing justice (see Table 4). Open-ended questions were utilized during the interviews to encourage participants to share detailed narratives, enabling the collection of rich qualitative data. Interviews with MDWs were conducted in their native language by current and former MDWs affiliated with Egna Legna Besidet's case management team, fostering trust and ensuring cultural and linguistic comfort for participants. Stakeholder consultations provided additional insights into institutional and systemic barriers affecting MDWs.

**Table 4:** Experiences of abuse and mistreatment.

**Category**	**Details**	**Number of participants**	**Percentage**
Sexual abuse	Experienced	17	25.76%
	Did not experience	41	62.12%
	Not reported	8	12.12%
Perpetrators of sexual abuse	Sponsor's son (once)	4	23.53% of those abused
	Sponsor's son (repeatedly)	2	11.76% of those abused
	Sponsor (once)	2	11.76% of those abused
	Sponsor (repeatedly)	2	11.76% of those abused
	Family member (once)	1	5.88% of those abused
	Family member (repeatedly)	5	29.41% of those abused
	Unknown street person	1	5.88% of those abused
Sexual harassment	Experienced	23	34.85%
	Did not experience	35	53.03%
	Not reported	8	12.12%
Perpetrators of sexual harassment	Sponsor (once)	1	4.35% of those harassed
	Sponsor (repeatedly)	4	17.39% of those harassed
	Sponsor's son (once)	3	13.04% of those harassed
	Sponsor's son (repeatedly)	5	21.74% of those harassed
	Family member (repeatedly)	5	21.74% of those harassed
	Taxi driver	1	4.35% of those harassed
	Unknown street person	1	4.35% of those harassed
Physical abuse	Experienced	37	56.06%
	Did not experience	26	39.39%
	Not reported	3	4.55%
Perpetrators of physical abuse	Male sponsor (repeatedly)	6	16.22% of those abused
	Female sponsor	9	24.32% of those abused
	Female sponsor (once)	2	5.41% of those abused
	Unknown street person	1	2.70% of those abused
	Family member (once)	2	5.41% of those abused
	Family member (repeatedly)	4	10.81% of those abused
	Family member and agency	4	10.81% of those abused
	Sponsor's children (once)	6	16.22% of those abused
	Sponsor's children (repeatedly)	3	8.11% of those abused
Verbal abuse	Experienced	54	81.82%
	Did not experience	7	10.61%
	Not reported	5	7.58%
Health issues	Experienced	24	36.36%
	Received medical care	5	20.83% of those with issues
	Did not receive medical care	18	75.00% of those with issues

Participants were selected purposively to ensure a diverse representation of experiences, capturing variations in age, duration of stay in Lebanon, and employment conditions. Recruitment was facilitated through Egna Legna Besidet's existing networks, ensuring access to a wide range of participant experiences. Data saturation was reached after 66 interviews, as no new themes or insights emerged, confirming the adequacy of the sample size for capturing the breadth of relevant experiences. The study employed thematic coding for data analysis, allowing for the identification of recurring patterns and themes. Using a grounded theory approach ensured that themes emerged organically from the data. The coding process was conducted manually and independently verified by two researchers to enhance reliability, while data triangulation through cross-referencing case files, interviews, and stakeholder feedback ensured the validity of the findings.

Ethical considerations were paramount throughout the research. The study received approval from the Institutional Review Board (IRB) at the Lebanese American University, and participation was entirely voluntary, with informed consent obtained from all participants. To safeguard confidentiality and anonymity, interview transcripts and translations were anonymized and coded, protecting the identities of MDWs and other informants. A trauma-informed approach was adopted during interviews to address the potential psychological impact of discussing sensitive or traumatic experiences (Diab and Al-Azzeh, 2024). Participants were also provided with information about support services in case the discussions evoked distress. The involvement of current and former MDWs in the interview process further ensured a safe and empathetic environment for participants to share their experiences.

The study was conducted over a period of 8 months, with 4 months allocated for data collection and an additional 4 months for data analysis, validation, and reporting. This phased approach allowed for thorough examination and cross-verification of data, ensuring the reliability and validity of the study's findings. By integrating diverse data sources and adopting a detailed, ethically sound methodology, the research provides a robust foundation for understanding the experiences and challenges faced by Ethiopian MDWs in Lebanon.

## 4 Theoretical framework

This study on the barriers to legal access faced by Ethiopian MDWs in Lebanon is grounded in three interconnected theoretical perspectives: intersectionality, structural violence, and legal pluralism. Together, these frameworks provide a comprehensive understanding of the multifaceted challenges these workers encounter.

Intersectionality, a concept introduced by Kimberlé Crenshaw, is essential for understanding the compounded discrimination experienced by Ethiopian MDWs. This framework examines how various forms of social stratification—such as race, gender, class, and nationality—intersect to create unique experiences of oppression and privilege (Crenshaw, 1991, 1989). Ethiopian MDWs in Lebanon occupy a particularly vulnerable position at the intersection of these identities, which exacerbates their susceptibility to exploitation, abuse, and legal marginalization. Intersectionality helps to reveal how these intersecting identities intensify the barriers to legal access, exposing the compounded nature of the injustices they face.

Johan Galtung's concept of structural violence provides insight into the systemic and institutionalized nature of the injustices faced by MDWs. Structural violence refers to social structures and institutions that harm individuals by preventing them from meeting their basic needs and achieving their full potential (Galtung, 1969; Vorobej, 2008; Burton et al., 2021). In the context of MDWs in Lebanon, structural violence manifests through legal systems, immigration policies, and socio-economic structures that perpetuate inequality and hinder access to justice. This perspective highlights how systemic factors, rather than direct physical violence, contribute to the exploitation and marginalization of MDWs. It emphasizes the role of institutional practices and socio-economic conditions in maintaining the status quo that disadvantages MDWs.

Legal pluralism, the coexistence of multiple legal systems within a single social field, is crucial for understanding the complexities of legal access for MDWs in Lebanon (Benda-Beckmann and Turner, 2018; Geoffrey, 2018). Lebanon's legal landscape includes formal state law, religious laws, and customary practices, each with its own norms and procedures. MDWs must navigate this plural legal system, where their rights and protections can vary significantly depending on the legal context in which their cases are adjudicated. Legal pluralism reveals how the interplay of different legal systems can create inconsistencies and contradictions that further complicate MDWs' access to justice. It highlights the challenges of obtaining fair treatment within a fragmented and overlapping legal framework.

By applying these theoretical perspectives, this study provides a nuanced understanding of the barriers to legal access faced by Ethiopian MDWs in Lebanon. Intersectionality reveals the compounded discrimination resulting from their intersecting identities. Structural violence uncovers the systemic forces that perpetuate their marginalization, while legal pluralism elucidates the complexities and contradictions within Lebanon's legal system that hinder their access to justice. Together, these theories offer a comprehensive lens through which to examine the challenges faced by MDWs and to propose informed and effective interventions. This integrated theoretical approach not only deepens the analysis of the injustices faced by MDWs but also informs recommendations for policy reforms and advocacy strategies. By addressing the systemic, intersectional, and legal dimensions of their experiences, this study aims to contribute to a more nuanced discussion around the development of an equitable and just legal landscape for MDWs in Lebanon.

## 5 Sample analysis

Unpacking the sample of those interviewed is crucial for understanding the study findings, as it provides nuanced insights into the diverse experiences of a falsely homogenized group of migrant domestic workers. By examining the positionality of each participant, the study acknowledges the various social, economic, and legal contexts that shape their experiences. Intersectional considerations, such as age, immigration status, type of journeys, and employment dynamics further illuminate how overlapping components of identity and lived experience contribute to unique vulnerabilities and challenges. This comprehensive approach ensures a more accurate and empathetic interpretation of the data, highlighting systemic issues and guiding more effective policy recommendations.

### 5.1 Notable demographic characteristics

Participants' ages at the time of migration varied significantly. Twelve women (18.18%) reported being underage, while the majority, 43 women (65.15%), were between 18 and 40 years old. Eight women (12.12%) did not provide age details, and 3 (4.55%) could not recall their age at migration. Discrepancies in reported passport ages were common: only 10 women stated their passport age was accurate, while 51 reported inaccuracies, typically listing an older age. One participant did not remember, and four did not provide an answer, highlighting documentation issues.

The time spent working in Lebanon ranged widely. One woman had been in Lebanon for < 2 years, 24 worked for under 5 years, 29 worked for under 10 years, and 9 were there for over 10 years. Three did not report their duration of stay.

Language proficiency revealed that 52 participants spoke Arabic, alongside their native Amharic. Two spoke only English, nine were proficient in both Arabic and English, and two were multilingual in Arabic, French, and English. Occupational status before migration varied: 25 were studying, 23 were working, four were both studying and working, one was neither, and six did not report their status. Educational levels also varied, with 4 having college degrees, none holding university degrees, 30 at the high school level, 15 at the elementary level, and 17 not reporting.

Participants migrated to Lebanon primarily for financial reasons, with others citing escape from domestic violence or forced marriages, family compulsion, or promises from recruitment agencies of better opportunities. The presence of social networks in Lebanon also influenced their decisions.

Out of 66 women, five had children while in Lebanon, 47 did not, and 14 did not answer. Of the five with children, three faced significant challenges obtaining legal documentation for their offspring, while two did not report their experiences.

### 5.2 Migration journeys and access to information

Travel arrangements for most participants were organized by recruitment agencies, with 50 women using this method. Four women had their travel arranged by family members, five by friends, and seven through other means. Payment for recruitment or placement fees varied: 28 paid fees in Ethiopia, four in a transit country, nine in Lebanon, and 18 in both Ethiopia and Lebanon. One woman paid fees in Ethiopia, a transit country, and Lebanon, while five paid no fees. Modes of entry into Lebanon also differed, with 41 women traveling directly by flight, nine through Sudan, five via Yemen, four through Dubai, and five using other routes. Two participants did not report their mode of entry.

Knowledge of recruitment agents was limited among participants. Only four women knew the name of their agent and agency, two forgot the name, two knew the Ethiopian agent or a family member of the agent, three did not respond, and 55 reported no knowledge of their recruitment agent. Agencies promised varying opportunities to participants, with some women being told about easy work, good salaries, or diverse jobs, while others received no information beyond travel dates.

Of the 24 participants who traveled through a transit country, 19 were not provided with a proper bed, two had proper beds, and three did not respond. Regarding abuse during transit, 21 reported no physical abuse, six experienced verbal abuse, 18 were not provided with sufficient food, one reported sexual harassment, and three did not provide an answer.

Upon arrival in Lebanon, many participants faced distressing conditions, including having their passports confiscated by agents, sponsors, or immigration police. Some were confined in small rooms without basic necessities and faced overcrowding and unsanitary conditions. However, a few women reported that their sponsors arrived at the airport on time. Work locations varied: 23 women worked in Beirut, 26 outside Beirut, seven did not respond, three worked in multiple locations, and eight did not know their work location.

Most participants were not briefed on their rights or duties. Only three women were informed about their rights, while 61 were not. Similarly, only four were informed about their duties and obligations by their employer, whereas 60 were not. Two participants did not provide an answer to either question. Contract translation was another significant challenge. Only three women reported that their contracts were translated into their native language before signing, while 60 stated that their contracts were not translated. Three participants did not answer.

### 5.3 Employment dynamics and payment

The reported salaries of participants reflect a concerning trend of substandard wages. Among the 66 participants, eight women earned < $150 per month, 46 were paid $150, seven received $200, three earned $250, and only one woman earned $400. One participant did not report her salary. Regarding payment from their first employer, only 13 women received full payment, while 47 did not, and four experienced non-payment from multiple employers. Two participants did not respond. Employers owed varying amounts to participants. Twenty-nine women were owed < 6 months' wages, 12 were owed less than a year's wages, four were owed < 2 years' wages, and three were owed < 3 years' wages. One woman was owed less than a year's wages from multiple employers, while seven women were fully paid. Ten participants did not report an answer.

Payment arrangements showed that 47 women had their wages sent directly to their families, two were paid directly in cash, and one reported payment to their agency. Sixteen participants did not specify their payment arrangements. Decisions regarding payment methods were predominantly controlled by sponsors (43 women), while only seven participants reported having autonomy in these decisions. Sixteen did not respond.

Living and working conditions were often inadequate. Nineteen women reported having sufficient food and water, while 46 did not. Thirteen women had their own bedroom, while 50 did not. Many participants without private sleeping spaces reported sleeping in unsuitable areas such as balconies, kitchens, or garages. Regarding privacy, only 10 women reported having privacy, while 51 did not. Participants worked excessively long hours, often between 14 and 21 h per day. Some were woken up at odd hours to continue duties, while others were required to do farm work or clean other homes, leading to insufficient sleep and illness. Many women performed additional tasks without extra compensation, including cleaning businesses or caring for family members in separate households.

Household sizes added to their workload: 32 women worked in households with 5–10 members, 24 in households with fewer than five members, and one in a household with more than 10 members. Fifty women reported working in multiple households. Thirty-six participants faced discrimination, including verbal abuse, religious intolerance, and denial of basic rights. Communication with family was restricted for many. While 48 women were allowed to communicate, 15 were not, and one was charged for phone calls. Four women were allowed to have their own phone, while 60 were not. Weekly day-offs were rare; only three women had them, while 63 did not. Similarly, five women were allowed to talk to others, whereas 61 were not. When it came to leaving the house, 11 women were permitted, while 54 were not.

### 5.4 Experiences of exploitation and abuse

Experiences of abuse were widespread among participants. Seventeen women reported being sexually abused, while 41 reported no such experiences, and eight did not provide an answer. The perpetrators varied: four women were abused once by their sponsor's son, two multiple times by the son, two once by their sponsor, two multiple times by their sponsor, one once by a family member, five many times by a family member, and 1 by an unknown person on the street.

Sexual harassment was reported by 23 women, while 35 reported no harassment, and eight did not provide an answer. Among those who experienced harassment, perpetrators included one sponsor who harassed a woman once, four sponsors who harassed women repeatedly, three sponsor's sons who harassed women once, five sponsor's sons who harassed women multiple times, five family members who harassed women repeatedly, one taxi driver, and one unknown person on the street.

Physical abuse was reported by 37 women, while 26 reported no abuse, and three did not answer. Perpetrators of physical abuse included six male sponsors who abused women multiple times, nine female sponsors, two female sponsors who abused women once, one unknown individual on the street, two family members who abused women once, four family members who abused women multiple times, four family members and agency staff, six sponsor's children who abused women once, and three sponsor's children who abused women repeatedly. Verbal abuse was the most common form of mistreatment, reported by 54 participants, with seven reporting no verbal abuse and five not responding. Health issues were prevalent, with 24 women reporting illnesses or accidents while working for their employers. Among these, only five received medical care, while 18 did not.

## 6 Analysis of findings

The findings from this study delve into the myriad obstacles hindering access to justice for this vulnerable demographic, shedding light on the profound legal violations they endure when prosecuted. By scrutinizing the initial investigation stage, trial proceedings, and the issuance of judgments, this section aims to unveil the systemic flaws perpetuating injustice and the magnitude of the legal injustices faced by MDWs within these critical stages of the legal process.

### 6.1 Experiences with legal entities and emergencies

Regarding the provision of guidance for dealing with emergencies or exposure to harm, only nine women reported receiving such guidance, while a significant majority, 46 women, did not receive any guidance. Eleven participants did not report an answer to this question. In terms of contacting their Lebanese or Ethiopian agent during emergencies, 14 women reported that they were able to make contact. However, 47 women were unable to reach their agents during emergencies, and five did not report an answer. This indicates a substantial gap in accessible support during critical situations.

When examining the helpfulness of agencies when contacted, the feedback was overwhelmingly negative. Only one woman reported that her agency was helpful. In contrast, 16 women found their agency to be unhelpful, and a substantial number, 48 women, did not provide an answer. This data highlights significant shortcomings in the support systems available to Ethiopian MDWs in Lebanon. Many workers did not receive guidance for emergencies, faced difficulties in contacting their agents during critical times, and often found their agencies unresponsive and unhelpful when assistance was sought. These findings underscore the urgent need for improved support mechanisms and reliable communication channels for MDWs in Lebanon.

A significant majority of the participants, 60 women, reported escaping from their employer's house. In contrast, two women did not escape, one woman was let go by her employer, two women were taken back to their agency by their employer, and one woman was arrested in her employer's house. Regarding incidents in the employer's home that required law enforcement or legal authorities' intervention, 44 women reported experiencing such incidents, while eight women said there were no such incidents. Fourteen women did not provide an answer.

The accounts of the challenges faced by workers who became undocumented because they escaped show that the lack of documentation made it exceedingly difficult to find and maintain stable employment, leading to periods of unemployment, exploitation, and abuse. Many were forced to accept lower wages or work without pay due to their vulnerable status. Undocumented status also prevented them from accessing essential medical care, with some avoiding treatment due to fear of discovery and others being denied services. The constant fear of being discovered by authorities limited the workers' movement and social interactions, while some experienced sexual harassment and assault, particularly in taxis or from their employers, with little recourse. Financial instability, isolation, depression, and anxiety were common experiences, compounded by language barriers and the inability to travel freely or return to their home countries. Ultimately, the workers felt they had no legal protection or means to seek justice, further entrenching their precarious and dehumanizing circumstances.

When it came to seeking justice, the majority of respondents who escaped the workplace reported taking no action, often due to fear of arrest, deportation, retaliation, or a belief that the system was biased against them. Some lacked information about their rights and available resources, while others felt that pursuing justice would be futile. A few respondents attempted to seek help, but were met with indifference or further mistreatment by the authorities. The participants' experiences with the Lebanese legal authorities, particularly the General Security, were overwhelmingly negative. Many felt that the authorities consistently sided with their employers without proper investigation, and that their legitimate complaints were ignored or dismissed. Several reported being treated “like criminals” despite being victims, with accounts of being mocked, yelled at, or denied the opportunity to defend themselves. None of the participants felt that their pursuit of justice met their expectations, indicating a unanimous dissatisfaction with the legal outcomes or processes they experienced.

In terms of seeking assistance from their embassy, 50 women reported requesting the embassy's assistance at some point, while 8 women did not seek such assistance. Eight participants did not report an answer. Among those who sought embassy help, only three women found the embassy to be helpful, whereas 48 women did not. Fifteen women did not report an answer, highlighting widespread dissatisfaction with embassy support. Many workers described feeling abandoned by the very institution meant to protect them. The embassy's failure to provide crucial information, support during legal issues, and adequate repatriation assistance left the workers feeling deeply betrayed and disillusioned. Some questioned the very existence or purpose of the embassy, with one woman asking rhetorically, “[…] does the Ethiopian embassy exist in Lebanon? No, they do not exist.” When it came to legal representation, 10 women reported that they were unable to attain it. In contrast, 32 women reported that they were able to attain legal representation or did not need it, and 24 participants did not report an answer.

Lastly, regarding outreach to NGOs and other entities for legal aid and assistance, 10 women reported that they had reached out for help, while 48 women had not. Seven participants did not provide an answer. This data highlights the severe challenges and dissatisfaction faced by Ethiopian MDWs in Lebanon, from escaping abusive environments to seeking justice and support from embassies and NGOs. The widespread dissatisfaction and lack of effective support underscore the urgent need for systemic reforms and better support mechanisms for these workers.

### 6.2 Enabling factors hindering adequate access

According to MDWs interviewed, an intertwined web of enabling factors contributes significantly to their inability to adequately access justice in Lebanon, first, as well as “be taken seriously” second. Alongside the structural issues attached to restrictive immigration policies and the Kafala sponsorship system, limited legal literacy as well as their isolation from public administrations, further hinder their ability to navigate the complex legal system, leaving them unaware of their rights or avenues for recourse.

Perhaps one of the most pivotal enabling factors is the selectivity in the application of the law. In Lebanon, while there are laws intended to protect the rights of everyone within its borders, including MDWs, the application and enforcement of these laws often fall short, leaving these vulnerable individuals without adequate protection. Law 205, which addresses sexual harassment, is a prime example (Diab and Al-Azzeh, 2024). While the law theoretically covers all individuals, including MDWs, its practical implementation faces numerous challenges. Many workers fear reporting instances of sexual harassment due to potential repercussions such as job loss, deportation threats, or further mistreatment by employers (Diab and Al-Azzeh, 2024). MDWs often lack awareness of their rights under this, and other laws. Even if they are aware, the obstacles to accessing legal recourse are immense, leading to underreporting and a lack of effective enforcement according to MDWs and experts interviewed. The authorities' limited enforcement of these laws, coupled with the complexities of legal procedures and the lack of access to legal representation for migrants, perpetuates a system where these individuals are systematically denied justice and fair treatment. Furthermore, the absence of specific provisions within the laws that account for the unique vulnerabilities and challenges faced by MDWs compounds their struggle for legal protection—as laws in Lebanon continue to fail in accounting for the diversity of individuals within the Lebanese territory (Diab and Al-Azzeh, 2024).

On their overall experiences with legal entities, Ethiopian women interviewed share harrowing tales of abuse, misconduct, racism and overall neglect at the hands of law enforcement officers and the legal system as a whole. Of the 66 women interviewed, 60 women stated that they had escaped from the homes of their employers. Reasons for running away included: (1) threats of inflicting harm or restricting freedom; (2) sexual assault, harassment, advances or inappropriate language and touching; (3) physical, emotional or psychological abuse; (4) lack of time off, privacy and unrealistic working hours; (5) lack of payment/reduction in salaries paid; (6) restricting communication with family, phone use and internet use; (7) entrapment in the house even during severe medical conditions; and (8) blatant racism, isolation and human rights violations.

Out of the 66 participants, 49 reported being arrested or having a criminal complaint filed against them, while only six had never faced such legal consequences. Notably, nine women were not arrested but had criminal complaints filed against them. Regarding the locations of the arrests, the findings reveal that nine women were apprehended at the airport, another nine were arrested in their employer's house, and one was detained at a police station. An additional 12 women were arrested in other unspecified locations, while 17 did not provide the location of their arrest.

According to in-depth interviews with respondents, one of the second most important components that enables their ill-treatment, as well as their limited access to justice across Lebanon, is the poor experiences they describe with their respective embassies and consulates in Lebanon—particularly, when they are “in trouble” with the law. Disturbingly, only 18 of the 49 participants who reported being arrested were allowed to contact their embassy or consulate after their arrest. A number of Ethiopian women interviewed describe sleeping outside their official government buildings for weeks on end to no avail—insisting that “[…] no one talked to us or helped us” (Interview, Beirut, 2023). Another Ethiopian participant expands on these negative experiences, even when there are health risks attached to their requests for support, insisting:

“[…] Eventually I went to the embassy when I got sick. I was really sick, and I told them I couldn't take it anymore. My stomach hurt, and possibly my kidneys too. They told me they didn't know what to do about that, and that I should go to the police. I asked them why I had to do that, why they couldn't help me, but they just said they couldn't help me and that I should go to the police myself, but I told them I didn't want to do that. They said I would have to wait for them to register my name and then I could go. After I was registered, they told me there was a charge made against me and I couldn't go back to Ethiopia” (Interview, Beirut, 2023).

Not only has the inability of MDWs to reach out to their embassies and consulates hindered their access to protection, but this has also placed them in a precarious situation with law enforcement in Lebanon, who according to a judge interviewed for this study, are “well aware” that MDWs do not have any safety nets or support system. She shares:

“[…] the reality is that law enforcement knows that our government is indifferent, their government is indifferent, and that the Lebanese legal system simply does not have the capacity, room or will power to support them. This has placed them in an ongoing perpetuated form of insecurity that cannot be resolved through the law at all. It is more structural, societal—and most importantly, it should be the priority of their respective governments to negotiate for them, protect their forms of labor and vouch for their citizens” (Interview, Beirut, 2023).

Ethiopian women interviewed highlight the incompetence and prolonged waiting periods attached to requests for support from their embassy, sharing:

“[…] I got registered at the embassy to travel, like other girls did during the COVID-19 pandemic, at the end of 2019. It took a really long time. 2021 was over and I was still not notified. For 2 years I wasn't granted a visa to travel, just coming and going” (Interview, Beirut, 2023).

Another Ethiopian woman shares:

“[…] I went and got registered at the embassy to travel back to Ethiopia. It took over 2 years. I used to go there a lot. The process took a long time. Every time I went there, they used to tell me ‘not yet”' (Interview, Beirut, 2023).

### 6.3 Initial investigation stage and trial

According to legal experts interviewed, when a complaint is filed against any person in Lebanon, the initial preliminary investigations are conducted by the judicial police, who act as assistants to the Public Prosecution within police stations. These investigations are conducted under the supervision of the competent Public Prosecution. This stage is crucial as it often forms the foundation upon which subsequent trials and judicial rulings are based. However, it is also a perilous stage where according to a legal expert interviewed for the purpose of this study, the suspect may be vulnerable to coercion and torture (Interview, Beirut, 2023). During initial investigations, the suspect enjoys fundamental rights outlined in Article 47 of the Code of Criminal Procedure (ALEF-Act for Human Rights, 2016). This article was amended pursuant to Law No. 191/2020, adding additional safeguards, particularly the right to seek the assistance of a lawyer during these proceedings (Legal Agenda, 2020).

Article 47 gives the right to any person accused to contact an attorney but it does not require counsel to be present during the investigative hearings (Legal Agenda, 2020). It also protects suspects or complainants if they refuse to speak or remain silent, and protects them from being compelled to speak or questioned under penalty of rendering their testimonies invalid (Legal Agenda, 2020). Other rights safeguarded under this article include: (1) the right to contact a lawyer of one's choice, a family member, an employer, or an acquaintance; and (2) the right to seek a lawyer's assistance during interrogation or statement hearings. Moreover, the investigating officer must inform the suspect or complainant of these rights before commencing the interrogation, record their position regarding utilization of these rights, and obtain their signature (Legal Agenda, 2020).

The suspect cannot be detained without a decision from the Public Prosecution and for a period not exceeding 48 h, which can be extended for a similar duration with the Public Prosecution's approval. The detention period starts from the time of detention. Following the expiration of the detention period, the Public Prosecution cannot take any action against the detained person. In addition to the aforementioned, the rights of the suspect during preliminary investigations further include: (1) ensuring confidentiality during the interview between the suspect and their lawyer, lasting a maximum of 30 min; (2) informing the suspect of the charges, suspicions against them, and supporting evidence to enable defense; (3) requesting a translator if the suspect is not fluent in Arabic; (4) requesting examination by a forensic doctor specializing in physical or mental health; (5) the right to be informed of their rights by judicial police before and after detention, failure of which invalidates the report; and (6) video recording interrogation procedures from the reading of the suspect's rights, attached to initial investigation minutes, under the risk of invalidation of records and subsequent procedures (Legal Agenda, 2020).

Despite the fact that Article 47 extends to MDWs, testimonies from Ethiopian participants outline a very different reality—and outline an overall environment of impunity throughout the legal process perpetuated by law enforcement officers. Out of the 49 women who reported being arrested, six experienced physical or verbal abuse at the hands of law enforcement officers, and one was physically assaulted by civilian men during her arrest. This abuse, combined with the widespread use of handcuffs (26 out of 49 women) and the overwhelming fear felt by 26 of the detainees during arrest, highlights the traumatic nature of the arrests. The detention conditions were equally appalling, with the women describing inadequate living spaces, poor quality and scarce food and water, lack of medical care, and widespread psychological distress. Many reported experiencing verbal abuse, physical violence, and discrimination from guards and other detainees, exacerbated by language barriers. As one respondent informs the study:

“I was afraid of them. I saw them in the prison. They don't have any discipline. They look at you in a wrong way, they yell at you, etc. They think all girls are the same. They all say the same things to us. They act like we are not humans. They didn't even let us talk to visitors who came to the prison. They made us wait outside when people wanted to talk to us. The ones who came running away from their employers were also forced to wait for hours outside. They would let us talk for only 10 min. Their treatment of us was not good at all. They have no manners. From the security to the police, no discipline” (Interview, Beirut, 2023).

While Article 14 of the International Covenant on Civil and Political Rights (ICCPR) to which Lebanon is signatory, guarantees a fair trial and safeguards against mistreatment and pressure, and while Article 47 mandates the right to appoint a lawyer before statements, and immediately after detention for investigation, testimonies from Ethiopian participants insist this has rarely been the case. Upon thorough and in-depth revision of more than 100 case files of preliminary investigations conducted with accused MDWs, emerging patterns reveal everything from a failure on the part of investigating officers to elaborate on the details of the rights outlined in Article 47, to failures and shortcomings in legal reporting throughout the preliminary investigation process, to the inability of MDWs' lawyers to request the nullification of statements based on these facts. Among the 49 participants who were arrested, a mere two had access to a translator in prison and 20 did not have access to a lawyer. Across a significant number of cases reviewed, police reports routinely stated that the MDWs being accused refused to benefit from the rights safeguarded by Article 47, and that they ultimately (and counter-intuitively according to a legal expert) refused legal representation, embassy assistance, or a translator. Particularly concerning is that these statements were also recorded in judicial files. As a lawyer interviewed for this study explains:

“[…] In many cases, the worker's refusal to appoint a lawyer may not be valid, especially if she does not comprehend Arabic and cannot verify the accuracy of the statement before signing it. Her refusal, if genuine, might be flawed due to her knowledge of the inability to communicate with or afford a lawyer's fees. In brief, how can we be sure that she exercised any form of agency and free will here?” (Interview, Beirut, 2023).

Based on study findings, in cases of arrest, when it came to being allowed visitors and the right to notify their family, only 11 women reported being granted these rights. In contrast, 17 women were not allowed visitors or the right to notify their family. The remaining 38 participants were either not arrested or charged or did not provide an answer. Out of the 49 research participants who were arrested, none were presented in court within 48 h of their arrest, highlighting a significant procedural failure. Regarding being informed of the reason for their arrest, only 18 women reported that they were told the reason, while 10 women were not informed, and 21 did not provide an answer.

When asked if they were given the opportunity to defend themselves in court, only six women reported having this opportunity. Fourteen women were not given the chance to defend themselves, three women did not go to court, and 34 participants did not provide an answer. None of the 58 women who were charged or arrested believed that they committed a crime worthy of arrest, indicating a perception of unjust treatment and wrongful charges. Among the 25 women who went to court, only 5 thought that their trial was fair, while 20 women did not believe their trial was fair. This reveals a significant lack of trust in the judicial process among the MDWs who participated in this study.

For this study, multiple case files reviewed, particularly ones overseen by the Tripoli Judicial Detachment, did not mention anywhere in reporting that Article 47 rights were read out loud to the MDW being prosecuted; even though reporting insisted that Article 47 “was considered.” Furthermore, the majority of the women who were arrested, 26 out of 49, reported that their rights as detainees were not explained to them. A lawyer interviewed for this study explains:

“[…] the issue here lies in the fact that because of the lack of supervision, professionalism, and an overall lack of humanity in approaching these cases, that there is no way of really knowing the extent to which Article 47 was explained or considered. These rights could be mentioned in passing, could be mentioned in sophisticated language that the MDW does not understand fully, and in many cases, could not be translated. When her lawyer wants to nullify the preliminary investigation, this complicates things for them, as it is their word against the word of the investigating officer” (Interview, Beirut, 2023).

Importantly, as per a lawyer consulted for this study, the Public Prosecution tends to pursue criminal cases based on suspicion (Interview, Beirut, 2023). For domestic workers, prosecutions often occur based on the sponsor's complaint without substantial evidence (Legal Agenda, 2020). Despite the Code of Criminal Procedure specifying a maximum detention period of 48 h during preliminary investigations, MDWs often face prolonged detention without legal justification (Global Detention Project, 2018). This extended detention, known as “administrative detention,” is more prevalent due to the vulnerable situation of these workers and the prevalence of legal violations against them (Global Detention Project, 2023). These violations extend beyond domestic workers and occur in various cases, indicating a broader systemic issue with arbitrary detention, more pronounced in marginalized groups, including women and sexual and gender minorities —a factor rendering MDWs intersectionally vulnerable. As a Gender Expert explains:

“[…] MDWs in Lebanon are part of a marginalized group because of the system that governs their forms of labor, their race, their gender as well as other socio-cultural considerations. When they are confronted with the discriminatory nature of Lebanon's legal system, this places them at a highly vulnerable intersection as women from this community who are basically silently overlooked by our legal protection frameworks” (Interview, Beirut, 2023).

The failure of several government agencies, in addition to the judicial system, further contribute to the gross human rights violations perpetrated against MDWs when they attempt to access any forms of legal support. The Ministry of Labor's failure to ensure that MDWs have been adequately paid according to their contracts has accumulated unpaid salaries owed by their employers (Amnesty International, 2020). According to a legal expert interviewed, the most common lawsuits against MDWs, leading to criminal rulings, are “absconding” and theft, often used by employers to evade their obligations (Interview, Beirut, 2023). Article 250 of the Code of Criminal Procedure states: “The trial shall be conducted orally. The President may decide to record it in audio or visual form. All evidence must be publicly discussed between the parties, with criminal material presented, and seizure records read. Each party may respond” (Police Human Rights Resources, 2001). How this plays out however, is an entirely different story. As an expert from the International Labor Organization (ILO) explains:

“[…] when it comes to MDWs, none of this happens. In many cases, these women are entirely isolated from the legal process, they do not understand the trial, and there is no translation provided. The right to a fair trial in-line with the International Covenant on Civil and Political rights is not upheld, nor are MDWs really informed of the stages of the proceedings, the possible outcomes, nor are they able to respond in many cases” (Interview, Beirut, 2023).

Under Article 14, the International Covenant on Civil and Political Rights (ICCPR) ensures: (1) equality before the judiciary; (2) the right to a fair, public trial by a competent, independent, impartial court; (3) the right to be considered innocent until proven guilty; as well as (4) ensures the accused enjoys minimum guarantees, including being informed promptly and in a language they understand, access to legal counsel, and a fair trial without undue delay (OHCHR, 1966). Despite this, court proceedings reviewed showcase prejudiced opinions on the part of judges and investigating officers. As a legal expert consulted explains:

“[…] judges should transcend stereotypes and biases, especially regarding domestic workers' perceived inferiority. They must not strip workers of their legal identity, such as misnaming or misidentifying them. The court must avoid bias toward employers and demand serious evidence to validate their statements. Serious violations by employers—like non-payment of wages, withholding residency papers, or restricting freedom—should be considered grounds for terminating employment and referred to the Public Prosecution upon verification”

He elaborates:

“[…] respecting the presumption of innocence is crucial. The court should not assume guilt during interrogation without solid evidence and should interpret doubts in the worker's favor, especially in cases with insufficient evidence where the employer's testimony is the primary basis. It's essential to acknowledge the unequal legal protection of the two parties involved in the trial” (Interview, Beirut, 2023).

### 6.4 Judgements issued and detention

Before presenting a sample of the rulings against MDWs in criminal cases where they are defendants, it is crucial to note that the current administrative framework pertaining to the legal status of foreigners, particularly the sponsorship system, primarily relies on administrative instructions and decisions issued by General Security. These instructions often lack proper legal grounding and remain largely unmonitored by legislative or judicial authorities. As per the law, every foreigner intending to work in Lebanon is required to obtain Ministry of Labor approval and a work visa from General Security (Tabar et al., 2020). They must submit an application within 10 days of entry to acquire a work permit from the Ministry of Labor and subsequently from General Security. Failure to renew residency within the stipulated period incurs penalties of imprisonment or fines. Employment changes mandate prior Ministry of Labor approval, subject to penalties as per the law; in some cases, General Security approval is also required, albeit unclear when. Conventionally, this intricately complex and ambiguous system has enabled the deportation of MDWs in non-stipulated cases, even while they are under prosecution, denying them fair trial rights (Mehzer et al., 2021). If a judgment contains discrepancies or violates fair trial principles, the convict retains the right to appeal using available legal methods; however, based on testimonies from Ethiopian participants, this has not always been the case.

Even more importantly, prolonged detention followed by an acquittal is a violation of international principles, warranting compensation for unlawful detention under Article 9 of the International Covenant on Civil and Political Rights. In application, this has also not been the case. As one participant shares:

“They detained me for a long time without informing me of their motives or reasons, and I still don't understand what they gained from my imprisonment. However, eventually, they told me to obtain a plane ticket and return to Ethiopia. It's absurd to think that throughout my entire time in prison, they only asked me to bring a flight ticket. Nevertheless, with the assistance of Egna Legna, I was able to successfully repatriate to my home country” (Interview, Beirut, 2023).

Moreover, there remains no legal provision for the defendant to seek compensation from the state in cases of detention leading to acquittal. While the Unified Employment Contract stipulates the employers' responsibility to procure and renew work permits and residency cards for the MDWs they employ, their avoidance of renewals has also left MDWs stuck in an irregular status without the means to rectify it. Should a MDW be fleeing an abusive employer's household with irregular status, this places her in heightened vulnerability of detention, coercion by authorities, or even as testimonies highlight, trafficking or smuggling.

As per an informant from the ILO, Lebanon signed ILO Convention No. 189 in 2011, focusing on decent work for domestic workers; however, the Lebanese Parliament has yet to ratify it (ILO Ministry of Labour, 2020). Despite international recommendations, Lebanon has not signed other significant conventions such as the International Convention No. 158, safeguarding migrant workers. The aforementioned has permitted for a rampant culture of impunity when it comes to MDWs, as well as a lack of adequate supervision of trials, detention, as well as rulings issued against them. Addressing various cases involving MDWs, one ruling by a single criminal judge in Baabda led to a defendant's conviction under the Foreigners Law and Decree No. 17561, resulting in a 3-month prison sentence, a fine, and deportation (Document Review, Beirut, 2023).

In a separate case in Beirut, a defendant faced imprisonment and charges for leaving her employer's home without renewing residence papers (Document Review, Beirut, 2023). Similarly, in Sidon, another defendant was acquitted of theft but convicted for leaving the employer's residence without consent, facing administrative regulation charges (Document Review, Beirut, 2023). Complaints to the General Directorate of General Security resulted in misdemeanor convictions for changing residence without notifying authorities and leaving the employer's home without prior consent (Document Review, Beirut, 2023). According to an informant who is a former IOM staff member, such cases are “the unfortunate norm” when it comes to the community. She elaborates:

“[…] these women [MDWs] face absolutely ridiculous charges, and are detained for some of the most illogical reasons. In many cases, they are detained for not having paperwork, a matter that is usually the result of their employer taking it away. In other cases, they are detained because they run away from an employer's household, when in most cases, she is fleeing an abusive working environment. We cannot approach MDWs' situations in a vacuum. There are so many contributing factors that need to be at play here, and need to be taken into consideration prior to conviction” (Interview, Beirut, 2023).

Article 12 of the International Covenant on Civil and Political Rights, to which Lebanon once again, is signatory, states: (1) Every individual lawfully within a state's territory has the right to freedom of movement within it and the freedom to choose their residence; (2) Everyone is free to leave any country, including their own; (3) These rights may only be restricted by laws necessary to protect national security, public order, public health or morals, or the rights and freedoms of others, consistent with the other rights in this Covenant; and (4) no one shall be arbitrarily deprived of the right to enter their own country. Despite Lebanon's international obligations, the majority of rulings, according to a legal expert consulted for this study,

“[…] followed a uniform pattern, suggesting they are like standardized templates, without the judge making the necessary effort to customize each ruling. The facts presented in the rulings were typically based solely on the account of the claimant, who is usually the sponsor or guarantor. There was a lack of sufficient evidence in several cases, leaving room for doubt. Despite this, the courts ruled for conviction.” (Interview, Beirut, 2023)

Amid these circumstances, detention and altercations with the law have had a profound and powerful impact on MDWs' mental health and fostered a pervasive sense of insecurity within the community. MDWs interviewed endure significant emotional distress when confronted with legal issues, detention, or deportation threats. The uncertainty surrounding their legal status amplifies feelings of anxiety, fear, and helplessness. Detention, separation from their support networks, and the looming possibility of deportation exacerbate their mental anguish, leading to what they describe as “psychological trauma” and “long-term emotional scars” (Interview, Beirut, 2023). Moreover, the broader MDW community, witnessing such instances, grapples with heightened apprehension and a profound sense of vulnerability, further fracturing their sense of safety and belonging within Lebanese society. As these incidents persist, the collective psyche within the community becomes burdened with an overwhelming sense of insecurity and unease, significantly impacting their overall wellbeing and sense of dignity. As one respondent shares: “[…] being detained impacted me mentally by causing distress, isolation, and emotional strain and was one of the worst experiences of my life” (Interview, Beirut, 2023).

Testimonies from participants who were detained highlight resorting to sleeping pills to sleep, an overall lack of access to medical attention even when they caught COVID-19, as well as fearing for their lives and fate without having any real prospects for a fair trial. For many respondents, their fate was completely unknown to them for months on end, until what many describe as a day where they were suddenly released, and ordered to return to Ethiopia. The uncertainty and lack of information surrounding the legal proceedings and release processes added to the women's suffering. While some were able to secure their release through legal means or with the assistance of advocacy organizations, many were simply informed they could leave without further explanation, often after serving their full sentences or facing deportation orders. Testimonies highlight abuse in detention at the hands of General Security, but also, at the hands of civil society organizations operating in these prisons, and who are intended to be there to support them. As a respondent who was detained with her daughter for several months describes:

“I was very sick and stressed. My daughter kept asking me when we will be going to Ethiopia. She wasn't sleeping well. A reputable organization was abusing us with GS. They get so much funding. They receive so many donations, but they are incompetent. They verbally abuse the people they are meant to help. They deprive them of basic human needs. It's laughable […] they would not even give us shampoo. I saw shampoo donations arriving. They give us floor detergent to wash ourselves with! I once washed my daughter with this detergent and she got a horrible rash. I asked them for medication to treat her skin, and they refused. This goes beyond deprivation of basic human needs. This is abuse” (Interview, Beirut, 2023).

The participants' testimonies reveal the profound and lasting impact their experiences in Lebanon have had on their lives, even after their return to Ethiopia. The women's accounts of the judges' decisions highlight the deep emotional distress and sense of injustice they felt. Many reported feeling sad, depressed, and even suicidal, with a strong perception that the judges were biased in favor of their employers. The lack of understanding regarding the legal proceedings, due to language barriers and inadequate explanations, further exacerbated their suffering.

The long-term physical and mental health consequences of their ordeals are equally concerning. Out of the 66 participants, 47 reported having trouble sleeping, and 45 had developed medical problems, underscoring the severe trauma they endured. The psychological impacts, including stress, anxiety, and a diminished sense of self-confidence, have significantly affected their ability to reintegrate into their communities. The reintegration process has been fraught with challenges, as many women faced disappointment, misunderstanding, and even mistreatment from their family members. Financial expectations and the inability to send home the anticipated remittances have led to tensions and conflicts within their households. Some chose to live separately from their families due to these issues.

Regarding their present circumstances, the interviewees have mixed experiences. While a few respondents reported positive experiences, such as pursuing education or finding stable employment, the majority described ongoing financial difficulties, lack of job opportunities, and continued health problems. The lasting impact of their time in Lebanon has left many feeling that their hopes for a better future have been shattered.

Notably, despite the overwhelmingly negative experiences, the majority of the women, 51 out of 66, reported feeling free upon their return to Ethiopia. This sense of freedom, often rooted in the ability to move freely and make their own decisions, underscores the fundamental importance of personal autonomy and the profound deprivation they faced in Lebanon. However, the financial burden they carry, with only 4 women reporting having money upon their return, has severely limited their ability to fully embrace this newfound freedom. The years spent in Lebanon, often without achieving their financial goals, have left many in a precarious state, struggling to rebuild their lives and support their families.

## 7 Discussion

This study provides a critical examination of the intersectional vulnerabilities faced by Ethiopian migrant domestic workers (MDWs) in Lebanon, significantly advancing our understanding of gender and the continuum of violence in migration. The findings confirm, contradict, and add nuance to the existing literature on labor migration, gendered violence, and structural inequality.

One of the key findings—the prevalence of underage migration, with 18.18% of participants reporting they were minors when they migrated—confirms prior research highlighting the vulnerabilities of young migrant workers in exploitative labor markets (Pande, 2013; Ghaddar et al., 2020). However, the data adds specificity by linking this to systemic failures in both Ethiopian and Lebanese regulatory frameworks, including inaccuracies in passport ages (reported by 77.27% of participants). This highlights a previously underexplored mechanism through which trafficking and exploitation are facilitated, advancing existing critiques of weak institutional protections for young female migrants.

The severe communication barriers faced by MDWs, including the denial of phone ownership (90.91%) and restricted family contact (72.73%), reflect the isolation tactics documented in other studies on domestic workers (Abdulrahim et al., 2024). However, the study builds on this literature by framing such restrictions as part of a broader continuum of psychological and emotional violence that perpetuates MDWs' dependency and vulnerability. These findings expand the understanding of how structural violence operates in intimate spaces, reinforcing previous studies that critique the Kafala system's role in creating exploitative conditions (Block et al., 2023; Pande, 2013; Dahdah, 2021).

The reported rates of physical abuse (56.06%), verbal abuse (81.82%), and sexual abuse (25.76%) align with existing research on gender-based violence in domestic work (Diab et al., 2023). Yet, this study highlights the intersectional dimensions of these abuses, showing how they are compounded by race, gender, and socio-economic inequalities. These findings confirm the systemic nature of exploitation identified in the literature but also emphasize the need for more rigorous enforcement of protective laws and employer monitoring, areas often neglected in policy discussions.

The Lebanese legal system emerges as a critical site of injustice, echoing findings from prior studies on the denial of due process and systemic bias against MDWs (Block et al., 2023; Pande, 2013; Abdulrahim et al., 2024). However, this study adds depth by illustrating the cumulative impact of legal pluralism in Lebanon, where state laws, religious codes, and customary practices create a fragmented and often contradictory legal landscape. This complexity exacerbates MDWs' inability to access justice, advancing theoretical discussions on the implications of legal pluralism in migration governance.

The study also challenges assumptions about recruitment agencies, showing not only their ineffectiveness but also their active role in perpetuating structural violence. While previous research has critiqued recruitment practices broadly (Fong, 2023), this study identifies specific gaps in oversight, such as the failure to brief workers on their rights and the lack of accountability during emergencies, adding actionable insights to policy debates.

In line with Johan Galtung's theory of structural violence, the study underscores how institutional frameworks perpetuate exploitation by denying MDWs access to basic protections and justice. Moreover, by integrating Kimberlé Crenshaw's intersectionality framework, it reveals how overlapping identities—gender, race, and migration status—compound these vulnerabilities. The study's application of legal pluralism further highlights the contradictions within Lebanon's legal systems, providing a more nuanced understanding of how MDWs are systematically excluded from justice.

Overall, this research confirms the structural inequalities and systemic barriers discussed in existing literature while adding new dimensions to the discourse on labor migration and gender. By combining empirical findings with theoretical insights, the study contributes to ongoing debates about the intersection of migration, justice, and gendered violence. These findings call for urgent reforms that not only address immediate legal protections but also dismantle the structural inequalities that perpetuate violence and exploitation.

## 8 Concluding remarks and recommendations

Addressing the challenges faced by Ethiopian MDWs in Lebanon requires a comprehensive and multi-faceted approach with specific responsibilities assigned to various stakeholders. Legal reforms are paramount to ensuring the protection and enforcement of MDWs' rights.

**To the Ministry of Interior (ISF and General Security)**: Children have been detained in adult detention facilities. It is imperative to coordinate with embassies and NGOs to establish safe spaces outside detention facilities to safeguard minors from inappropriate conditions. Domestic workers, especially live-in workers detained from their sponsor's house, often have no access to legal assistance and are burdened with court fees, penalties, and other costs they cannot cover. The police must promptly communicate with relevant CSOs and embassies to ensure services are provided for detainees. In cases of false accusations, employers should cover the workers' release expenses.

Many domestic worker detainees do not speak Arabic, preventing them from understanding the charges against them or defending themselves during interrogations. Police should coordinate with embassies and NGOs to provide interpreters to ensure detainees are well-informed during legal proceedings. Additionally, migrant domestic workers are often detained without evidence, sometimes as a favor to friends or family, leading to common physical and verbal abuse during arrests. Human rights training for ISF and General Security personnel is essential, along with implementing accountability mechanisms for unlawful arrests. After obtaining visas and air tickets, MDWs are often arrested at the airport due to pending complaints, spending months detained without notifying families or embassies. Immediate notification of embassies and allowing detainees to contact their families is crucial. Establishing a monitoring and referral mechanism within the Ministry of Interior to centralize data and issue public reports on MDWs' status is necessary.

**To the Judiciary**: Unlawful detention and lack of compensation for wrongly detained MDWs are significant issues. Sponsors who make false accusations must be held accountable and cover compensation, legal, and repatriation fees. Judges should receive comprehensive training in international human rights laws, treaties, and mechanisms and specialized training in immigration and Kafala procedures. Establishing specialized chambers within the Lebanese Judicial system for immigration-related cases and ensuring detainees can change their lawyer if needed are critical steps. Facilitating access to complaints and appeals mechanisms for MDWs can hold sponsors accountable for false accusations.

**To Embassies**: Embassies need to coordinate with NGOs to provide safe spaces for children to prevent them from being detained in adult facilities. They must offer interpreters to ensure MDWs understand their charges and can defend themselves in court. Establishing protocols for prompt notification and communication with relevant authorities when MDWs are detained is vital. Embassies should work closely with local NGOs to provide legal aid, interpreters, and other support services, ensuring MDWs know their rights and available support in Lebanon.

**To Civil Society Organizations**: CSOs should establish partnerships with embassies and Lebanese authorities to facilitate access to detained MDWs and provide necessary support services, including legal aid and translation services. Advocacy for the rights of detained MDWs is crucial, as is campaigning for improved detention conditions and fair legal processes. Conducting training and awareness programs for law enforcement and judiciary on MDWs' rights and due process is essential. Collaborating with embassies to provide safe spaces for MDWs fleeing abuse or awaiting legal proceedings is also necessary.

In summary, this study highlights the pressing need for a concerted effort to implement comprehensive reforms and ensure greater protection for Ethiopian MDWs in Lebanon. By addressing systemic and structural issues and assigning clear responsibilities to various stakeholders, we can work toward upholding the rights and dignity of MDWs. These measures will not only enhance legal and social protections but also foster a more inclusive and respectful environment for all workers.

## Data Availability

The raw data supporting the conclusions of this article will be made available by the authors, without undue reservation.

## References

[B1] Abdulrahim S. Cherri Z. Adra M. Hassan F. (2024). Beyond Kafala! Employers' discriminatory attitudes and violations of the rights and freedoms of women migrant domestic workers in Lebanon. Migrat. Stud. 12:mnae012. 10.1093/migration/mnae012

[B2] ALEF-Act for Human Rights (2016). The Right to Fair Trial in Lebanon A Position Paper on Guarantees During Arrest and Investigation. ALEF Liban. Available at: https://alefliban.org/wp-content/uploads/2016/10/PP1_v02_print.pdf (accessed June 15, 2024).

[B3] Amnesty International (2019). End Kafala: Justice for Migrant Domestic Workers in Lebanon. Available at: https://www.amnesty.org/en/latest/campaigns/2019/04/lebanon-migrant-domestic-workers-their-house-is-our-prison/ (accessed June 15, 2024).

[B4] Amnesty International (2020). Lebanon: Abandoned Migrant Domestic Workers Must Be Protected. Available at: https://www.amnesty.org/en/latest/news/2020/06/lebanon-abandoned-migrant-domestic-workers-must-be-protected/ (accessed June 15, 2024).

[B5] Barakat J. (2023). Lebanon: Honorary Consuls Exploiting Women Migrant Workers. Daraj Media. Available at: https://daraj.media/en/lebanon-honorary-consuls-exploiting-women-migrant-workers/ (accessed June 15, 2024).

[B6] Benda-Beckmann K. V. Turner B. (2018). Legal pluralism, social theory, and the state. J. Legal Plural. Unoff. Law 50, 255–274. 10.1080/07329113.2018.1532674

[B7] Block K. Fernandez B. McGee T. Al-Barazi Z. Brennan D. (2023). Immobilisation of migrant domestic worker women and their children born in Lebanon. J. Ethnic Migrat. Stud. 50, 1923–1940. 10.1080/1369183X.2023.2245153

[B8] Bondakji J. (2021). Racism and the Horrific Treatment of Migrant Workers in Lebanon. Femonomic. Available at: https://femonomic.com/racism-and-the-horrific-treatment-of-migrant-workers-in-lebanon/ (accessed June 15, 2024).

[B9] Burton C. W. Gilpin C. E. Draughon Moret J. (2021). Structural violence: a concept analysis to inform nursing science and practice. Nurs. For. 56, 382–388. 10.1111/nuf.12535 33355920

[B10] Crenshaw K. (1989). Demarginalizing the intersection of race and sex: a black feminist critique of antidiscrimination doctrine, feminist theory and antiracist politics. Univ. Chicago Legal For. 1989:8.

[B11] Crenshaw K. (1991). Mapping the margins: intersectionality, identity politics, and violence against women of color. Stanford Law Rev. 43, 1241–1299. 10.2307/1229039

[B12] Dahdah A. (2021). The familiar tune of refugee, migrant, and displaced worker exploitation in the Lebanese labour market. Revue européenne des migrations internationales 37:18275. 10.4000/remi.18275

[B13] Diab J. L. Al-Azzeh D. (2024). Inclusive inquiry: a compassionate journey in trauma-informed qualitative research with GBV survivors from displaced communities. Front. Psychol. 15:1399115. 10.3389/fpsyg.2024.1399115 39118846 PMC11307779

[B14] Diab J. L. Yimer B. Birhanu T. Kitoko A. Gidey A. Ankrah F. (2023). The gender dimensions of sexual violence against migrant domestic workers in post-2019 Lebanon. Front. Sociol. 7:1091957. 10.3389/fsoc.2022.1091957 36741584 PMC9891457

[B15] Ebert B. (2015). Institutional Consensus: A Comparative Analysis of Rules of Law in Lebanon and Somalia. Sound Ideas; Student Research and Creative Works. University of Puget Sound. Available at: https://jstor.org/stable/community.36514247 (accessed June 15, 2024).

[B16] Edwards M. Sousa J. (2024). Lebanon's Migrant Workers Left Stranded and Homeless By Israeli Attacks: “We Are Totally Abandoned.” The New Humanitarian. Available at: https://www.thenewhumanitarian.org/news-feature/2024/09/26/lebanons-migrant-workers-left-stranded-homeless-israeli-attacks (accessed June 15, 2024).

[B17] El Hage A.-M. (2023). “*No Excuses for Abuse and Violence”: Migrant Domestic Workers Air Frustrations at Beirut Demonstration*. L'Orient Today. Available at: https://today.lorientlejour.com/article/1336035/no-excuses-for-abuse-and-violence-migrant-domestic-workers-air-frustrations-at-beirut-demonstration.html (accessed June 15, 2024).

[B18] Fong E. (2023). Review of “unfree: migrant domestic work in Arab States.” *Social Forces* 101:e9. 10.1093/sf/soac145

[B19] Galtung J. (1969). Violence, peace, and peace research. J. Peace Res. 6, 167–191. 10.1177/002234336900600301

[B20] Geoffrey S. (2018). Legal pluralism in theory and practice. Int. Stud. Rev. 20, 438–462. 10.1093/isr/vix060

[B21] Ghaddar A. Khandaqji S. Ghattas J. (2020). Justifying abuse of women migrant domestic workers in Lebanon: the opinion of recruitment agencies. Gaceta Sanitaria 34, 493–499. 10.1016/j.gaceta.2018.11.001 30594331

[B22] Global Detention Project (2018). Country Detention Report: Immigration Detention in Lebanon: Deprivation of Liberty at the Frontiers of Global Conflict. Available at: https://www.globaldetentionproject.org/wp-content/uploads/2018/02/GDP-Immigration-Detention-Report-Lebanon-2018-1.pdf (accessed June 15, 2024).

[B23] Global Detention Project (2023). Lebanon. Available at: https://www.globaldetentionproject.org/countries/middle-east/lebanon (accessed June 15, 2024).

[B24] ILO (2014). Migrant Domestic Workers Lack Adequate Access to Justice in Lebanon. International Labour Organization. Available at: https://www.ilo.org/resource/news/migrant-domestic-workers-lack-adequate-access-justice-lebanon (accessed June 15, 2024).

[B25] ILO and Ministry of Labour (2020). National Consultation on Kafala Reform in Lebanon. Available at: https://www.ilo.org/sites/default/files/wcmsp5/groups/public/@arabstates/@ro-beirut/documents/meetingdocument/wcms_737980.pdf (accessed June 15, 2024).

[B26] Karam P. (2024). The Limits of Justice in Lebanon—and What It Means for the Future of the Country. Washington, DC: Arab Center. Available at: https://arabcenterdc.org/resource/the-limits-of-justice-in-lebanon-and-what-it-means-for-the-future-of-the-country/ (accessed June 15, 2024).

[B27] Legal Agenda (2020). Legal Agenda Statement on Law No. 191/2020: A Call to Adhere to the Principles of Fair Trial and Judicial Independence. Available at: https://legal-agenda.com/%D8%A8%D9%8A%D8%A7%D9%86-%D8%A7%D9%84%D9%85%D9%81%D9%83%D8%B1%D8%A9-%D8%AD%D9%88%D9%84-%D8%A7%D9%84%D9%82%D8%A7%D9%86%D9%88%D9%86-%D8%B1%D9%82%D9%85-191-2020-%D8%AF%D8%B9%D9%88%D8%A9-%D9%84/ (accessed June 15, 2024).

[B28] Malaeb B. (2018). State Fragility in Lebanon: Proximate Causes and Sources of Resilience. LSE-Oxford Commission on State Fragility, Growth and Development. Available at: https://www.theigc.org/sites/default/files/2018/04/Lebanon-country-report.pdf (accessed June 15, 2024).

[B29] Mansour M. W. Daoud C. Y. (2010). Lebanon: The Independence and Impartiality of the Judiciary. Euro-Med Human Rights Network. Available at: https://euromedrights.org/wp-content/uploads/2018/03/LEBANON-The-Independence-and-Impartiality-of-the-Judiciary-EN.pdf (accessed June 15, 2024).

[B30] Mehzer Z. Nassif G. Wilson C. (2021). Migrant Women's Rights are Women's Rights. Women Migrant Domestic Workers in Lebanon: A Gender Perspective. Arab Institute for Women, Lebanese American University, IOM, ILO, UN Women. Available at: https://mena.iom.int/sites/g/files/tmzbdl686/files/documents/Migrant%20Workers%27%20Rights%20are%20Women%27s%20Rights_%20June%2016%20_%202021%20FINAL_0.pdf (accessed June 15, 2024).

[B31] Nasri A. Tannous W. (2017). Access to Justice for Migrant Domestic Workers in Lebanon. International Labour Organization with Caritas Lebanon. Available at: https://humantraffickingsearch.org/wp-content/uploads/2017/07/access-to-justice.pdf (accessed June 15, 2024).

[B32] OHCHR (1966). International Covenant on Civil and Political Rights. United Nations Human Rights Office of the High Commissioner. Available at: https://www.ohchr.org/en/instruments-mechanisms/instruments/international-covenant-civil-and-political-rights (accessed June 15, 2024).

[B33] Pande A. (2012). From “balcony talk” and “practical prayers” to illegal collectives: migrant domestic workers and meso-level resistances in Lebanon. Gender Soc. 26, 382–405. 10.1177/0891243212439247

[B34] Pande A. (2013). ‘The paper that you have in your hand is my freedom': migrant domestic work and the sponsorship (Kafala) system in Lebanon. Int. Migrat. Rev. 47, 414–441. 10.1111/imre.12025

[B35] Pande A. (2018). Intimate counter-spaces of migrant domestic workers in Lebanon. Int. Migrat. Rev. 52, 780–808. 10.1111/imre.12325

[B36] Police and Human Rights Resources (2001). Act No. 328 of 7 August 2001 as amended by Act No. 359 of 16 August 2001. Available at: https://policehumanrightsresources.org/content/uploads/2016/07/Criminal-Procedure-Code-Lebanon-2001.pdf?x54919 (accessed June 15, 2024).

[B37] Rowitz L. (2023). The Standard Unified Contract for Migrant Domestic Workers in Lebanon — Recognition of Rights and Responsibilities or Facilitation of ‘Modern Slavery'? Trasis Blog. Universität Bern. Available at: https://trasisblog.unibe.ch/?p=208 (accessed June 15, 2024).

[B38] Saghieh N. (2020). Lebanon's New Standard Domestic Worker Contract: A Coin with Two Sides. The Legal Agenda. Available at: https://english.legal-agenda.com/lebanons-new-standard-domestic-worker-contract-a-coin-with-two-sides/ (accessed June 15, 2024).

[B39] Sater J. (2013). “Migrant workers, labor rights, and governance in middle income countries: the case of migrant domestic workers in Lebanon,” in Migration, Security, and Citizenship in the Middle East. The Modern Muslim World, eds. P. Seeberg and Z. Eyadat (New York, NY: Palgrave Macmillan), 6.

[B40] Tabar P. Denison A. Alkhomassy M. (2020). “Access to social protection by immigrants, emigrants and resident nationals in Lebanon,” in Migration and Social Protection in Europe and Beyond (Volume 3). IMISCOE Research Series, eds. J. M. Lafleur and D. Vintila (Cham: Springer), 10.

[B41] The Legal Agenda and ILO (2020). The Labyrinth of Justice: Migrant Domestic Workers Before Lebanon's Courts. Available at: https://www.fairrecruitmenthub.org/sites/default/files/2023-10/wcms_777078_0.pdf (accessed June 15, 2024).

[B42] Vorobej M. (2008). Structural violence. Peace Res. 40, 84–98. Available at: https://www.jstor.org/stable/23607799

